# Advancements and current trends in tumor treating fields: a scientometric analysis

**DOI:** 10.1097/JS9.0000000000001151

**Published:** 2024-02-13

**Authors:** Yang Xing, Feroza Yasinjan, Jiayue Cui, Yizhao Peng, Minghua He, Wenhui Liu, Xinyu Hong

**Affiliations:** a Department of Neurosurgery, The First Hospital of Jilin University; b Department of Histology and Embryology, College of Basic Medical Sciences; c College of Computer Science and Technology, Jilin University, Changchun, People’s Republic of China

**Keywords:** GBM, glioma, scientometric analysis, TTfields, tumor treating fields

## Abstract

Tumor treating fields (TTFields) therapy is a novel and effective noninvasive cancer therapy, and it has been approved by FDA in the treatment of recurrent and newly diagnosed glioblastoma, and malignant pleural mesothelioma. Moreover, TTFields therapy has been widely studied in both clinical trials and preclinical studies in recent years. Based on its high efficacy, research on TTFields therapy has been a hot topic. Thus, the authors made this scientometric analysis of TTfields to reveal the scientometric distributions such as annual publications and citations, countries and institutions, authors, journals, references, and more importantly, research status and hot topics of the field. In recent years, publication numbers have been stable at high values, and citation numbers have been increasing greatly. The United States and Israel were the top two countries with the highest publication numbers, followed by Germany and Switzerland. Scientometric analyses of keywords indicated that clinical applications and antitumor mechanisms are probably the two main parts of current research on TTfields. Most clinical trials of TTfields focus on the treatment of glioblastoma. And a variety of other cancers such as lung cancer especially nonsmall cell lung cancer, hepatic cancer, other brain tumors, etc. have also been studied in both clinical trials and preclinical studies.

## Introduction

Highlights

- This study represents the first scientometric analysis specifically focused on Tumor treating fields (TTFields) in all domains.
- Through a comprehensive scientometric analysis, the current research situations, hotspots, and emerging trends in the TTFields domain are thoroughly mapped out.
- The study accentuates the clinical applicability of TTFields, particularly in glioblastoma, as well as its antitumor mechanisms, offering a dual perspective on its significance.

Tumor treating fields (TTFields) therapy is a novel noninvasive cancer therapy, which exerts the antitumor effect based on the alternating electric fields applied at low-intensity (1–3 V/cm) and intermediate-frequency (100–500 kHz)^1^. In 2011, FDA approved TTFields therapy for the treatment of recurrent or refractory glioblastoma, based on the active results of EF-11 clinical trials (NCT00379470)^2^. Then in 2015, FDA approved TTFields therapy for the treatment of newly diagnosed glioblastoma, based on the excellent results of EF-14 clinical trials (NCT00916409)^3^. Compared with single temozolomide, TTFields therapy plus temozolomide can help extend the overall survival (OS) and progression-free survival (PFS) of patients with newly diagnosed glioblastoma to approximately five and three months, respectively (PFS, 7.1 vs 4.2 months) and OS (20.5 vs 15.6 months)^4^. It is worth noting that TTFields therapy plus temozolomide is the first newly approved treatment regime by FDA for newly diagnosed glioblastoma in the past 10 years. In 2019 NovoTTF-100L system in combination with Pemetrexed and platinum-based chemotherapy was approved by FDA for the first-line treatment of locally advanced or metastatic malignant pleural mesothelioma (MPM) that is inoperable, based on a phase 2 trial STELLAR^5^. Similarly, this approval is the first newly approved treatment regime by FDA for MPM in the past 15 years. In 2021, FDA granted breakthrough device designation to the NovoTTF-200T system to use together with Atezolizumab and Bevacizumab for the treatment of advanced liver cancer, partly based on the results from the HEPANOVA phase II study^6^. These approvals or grants have strongly indicated that TTFields therapy is a promising and efficient tool to combat with cancers. Moreover, TTFields therapy alone or combined with other therapies have also been widely explored in other clinical trials for treating various cancer including lung cancer^7,8^, pancreatic cancer^9,10^, breast cancer^11,12^, ovarian cancer^9,13^, etc.

Besides the active attempts in the clinical trials, many studies also explore the antitumor mechanisms of TTFields therapy^14,15^. It is widely acknowledged that the main target of TTFields is mitosis and division of cancer cells^16^. Besides, other mechanisms such as interruption of DNA damage response, disruption of cellular components have also been studied^16^. It is worth noting that exploring and clarifying detailed antitumor mechanisms is useful for combined therapies to enhance antitumor effects^17^.

In the realm of scientific inquiry, scientometrics plays an increasingly pivotal role in delineating research landscapes, pinpointing areas of concentrated scholarly activity, and discerning emergent trends by rigorously analyzing existing literatures across specific disciplines^18–22^. Given the accelerating advancements in the utilization of TTFields for oncological interventions, it becomes imperative to delineate the contours of research milestones, focal points, and evolving paradigms within this domain. Such an endeavor not only offers valuable insights to the scientific community but also propels the field toward innovative pathways, thus enhancing the therapeutic arsenal against malignancies.

## Materials and methods

### Data source and searching criteria

For the purposes of conducting a robust scientometric analysis, we elected to use the Science Citation Index Expanded (SCIE), housed within the Web of Science Core Collection (WoSCC), as our primary database for literature retrieval. This platform is recognized for its comprehensive and exhaustive aggregation of scientific literature across multiple disciplines. The search parameters were devised to ensure maximal specificity and relevance to the topic under investigation, and were operationalized as follows:

(1) Search term: Topic (TS) encompassed both ‘tumor treating fields’ and its commonly used acronym, ‘TTFields’.

(2) Document types: The search was limited to meeting Abstracts, Articles, and Review Articles to ensure academic rigor and subject relevance.

(3) Language: Only manuscripts published in English were considered for inclusion in this analysis.

**Figure 1:**
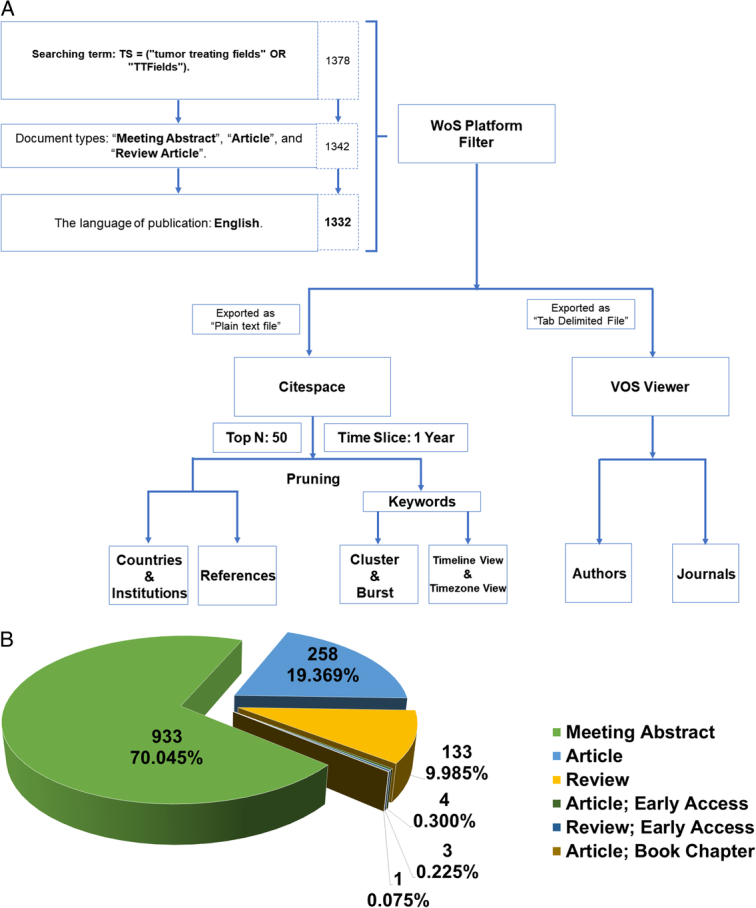
Overview of study methodology and document classifications. (A) Flowchart delineating the inclusion criteria and research steps. (B) Categorical distribution of document types, displayed in percentage.

The search was executed on 23 September 2023, culminating in the identification of 1332 records that met the aforementioned criteria. A flow diagram elucidating the methodology employed in this study is presented in Figure 1A.

### Data analysis and methodology

CiteSpace serves as a potent instrument for conducting both interactive and exploratory analyses across scientific knowledge domains^23^. The software employs a hybrid approach that integrates both qualitative and quantitative methodologies, enabling the dissection of knowledge structures and the identification of research focal points and trends^24^. Upon exporting the 1332 records from Web of Science (WoS) to CiteSpace in plain text format and after eliminating duplicate entries, we were able to determine the distribution of document types: 933 meeting abstracts (70.05%), 263 articles (19.74%), and 136 reviews (10.21%). The operational parameters within CiteSpace were configured as follows:

(1) Time slices spanned from January 2006 to December 2023, each slice corresponding to a single calendar year.

(2) Selection criteria were designed to highlight the top 50 most-cited or most-occurring items within each time slice.

(3) Pruning functions deployed included pathfinder, pruning of sliced networks, and pruning of the merged network.

In the analysis, CiteSpace facilitated an examination of research-producing countries and institutions, reference clustering, timeline and timezone views, as well as keyword burst analysis.

In contrast, VOS Viewer emphasizes the graphical visualization of scientometric data, offering a streamlined platform for constructing and interpreting large visualization maps^25^. The 1332 records were exported from WoSCC to VOS Viewer as tab-delimited files. Initially, we opted to ‘create a map based on bibliographic data’, followed by the selection to ‘read data from bibliographic database files’. Subsequent to the data import, we engaged in selective scientometric analysis.

Within the framework of this study, VOS Viewer was employed to scrutinize the contributions and impact of authors and academic journals in the domain of TTFields. By employing both CiteSpace and VOS Viewer, this study seeks to offer a composite, multidimensional analysis, thus enriching our understanding of the current landscape and future directions in the realm of TTFields in oncological treatment.

## Results

### Annual distributions of publications and citations

**Figure 2:**
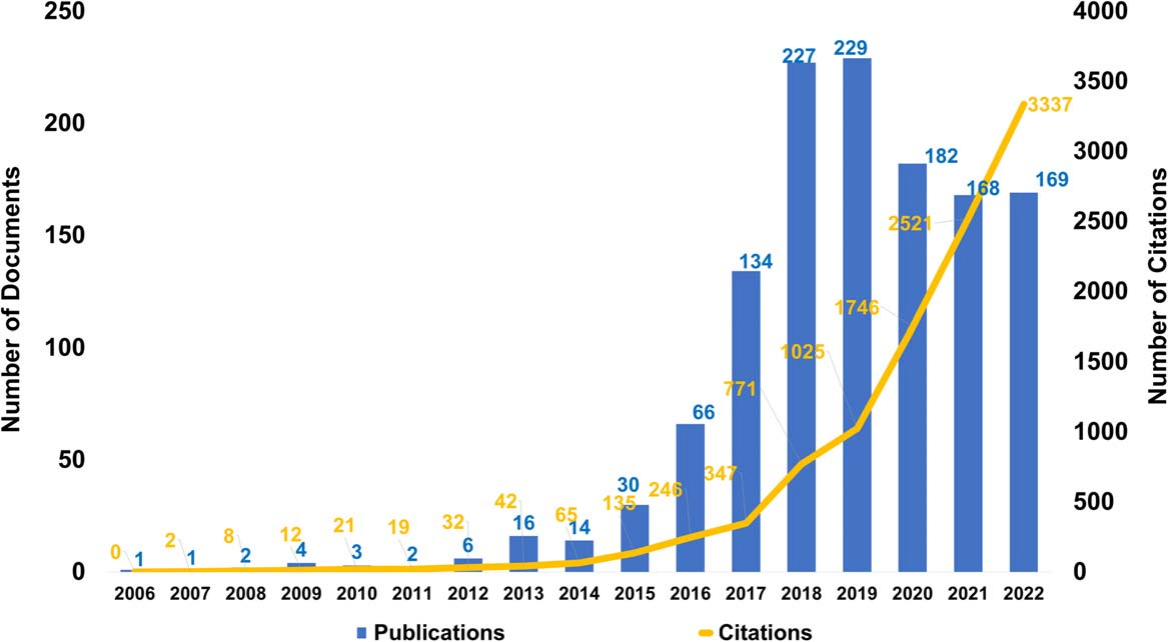
Temporal trends illustrating annual publication counts and their corresponding citations in the field of TTFields.

The temporal patterns of publications and citations in the realm of TTFields were scrutinized for the period extending from 2006 to 2023 (Fig. 2). Within this time frame, a corpus of 1332 publications was amassed, garnering a cumulative citation count of 12 398. This equates to an average citation frequency of ~9.31 citations per paper. A nuanced view of the data reveals several pivotal inflection points. Notably, the years preceding 2014 were characterized by a sparse volume of publications related to TTFields, thereby suggesting a period of nascent scholarly interest. However, an inflection was observed in the period from 2015 to 2019, where there was a conspicuous escalation in the rate of annual publications. Post-2020, the tempo of annual publications appeared to reach a plateau, maintaining relative stability. In terms of citation metrics, the trajectory displayed a moderate incline from 2006 to 2014. Subsequently, a more accelerated and steady uptick in citation counts was recorded. Projections based on these trends suggest that while the volume of publications may plateau in the foreseeable future, the citation count is likely to persist in its upward trajectory. Collectively, these observations underscore the mounting academic and clinical interest in TTFields, indicative of its emergent prominence as a subject of substantive inquiry and its potential therapeutic utility in the field of oncology.

### Distributions of countries and institutions

**Figure 3:**
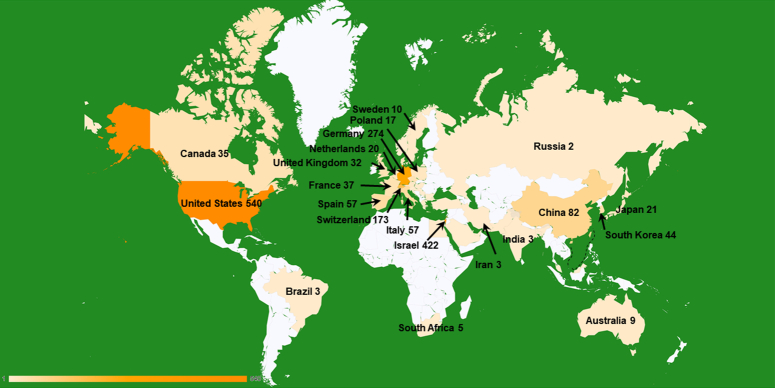
Heat map representing the geographical dispersion of research publications in TTFields by country.

The body of literature on TTFields originates from a diverse array of 46 countries and regions, each contributing at least one relevant publication to the field (Fig. 3, Table S1, Supplemental Digital Content 1, http://links.lww.com/JS9/B930). Among all the countries, the United States had an overwhelmingly high publication number (*n*=542, accounting for 40.54%). Israel ranked second, with 422 relevant publications in this field. Other countries with high publication numbers included Germany (*n*=274, 20.57%), Switzerland (*n*=173, 12.99%), China (*n*=78, 5.86%), Spain (*n*=57, 4.28%), and Italy (*n*=57, 4.28%). Three countries including Israel, Switzerland, and Czech Republic had the earliest publication in 2006. It is worth noting that many countries such as Germany, China, Spain, South Korea, Canada owned their first publications after 2015, indicating the emerging role of TTFields. Countries with high betweenness centralities were also identified, including France (betweenness centrality (bc)=1.01), Italy (bc=0.95), Czech Republic (bc=0.67), Israel (bc=0.49), the United States (bc=0.47), representing their high influences in this field^26^.

Institutional participation in the field of TTFields is both diverse and extensive, with a total of 369 institutions contributing at least one publication. Within this cohort, 28 institutions have produced more than 20 publications each, while 64 institutions have contributed upwards of ten publications. Remarkably, Novocure—a pioneering oncology company based in Israel—stands at the zenith of this landscape with an impressive 405 publications. As an early leader in TTFields for solid tumors, Novocure also holds the distinction of being the first institution to publish in this domain. Following Novocure, the University of Texas System occupies the second position with 70 publications, trailed by Tel Aviv University (*n*=64), University of Duisburg Essen (*n*=60), Harvard University (*n*=59), and Sackler Faculty of Medicine (*n*=52). In terms of geographical distribution, American institutions are significantly represented, making up 11 of the top 20 high-publishing organizations. Additionally, five institutions from Israel and four from Germany also appear on this list, thereby delineating the United States, Israel, and Germany as the principal geographical epicenters for TTFields research and development. Collectively, these data illuminate not only the leading institutional contributors but also highlight the geopolitical landscapes where TTFields are being intensively researched and potentially applied in oncological treatment paradigms (Table 1).

**Table 1:** The top 20 institutes with the most publications in this field.

No.	Institutes	Counts	Begin years
1	Novocure (Israel and the United States)	405	2006
2	University of Texas System (the United States)	70	2014
3	Tel Aviv University (Israel)	64	2012
4	University of Duisburg Essen (Germany)	60	2018
5	Harvard University (the United States)	59	2013
6	Sackler Faculty of Medicine (Israel)	52	2012
7	Beth Israel Deaconess Medical Center (Israel)	43	2013
8	University of Wurzburg (Germany)	40	2018
9	University of California System (the United States)	39	2012
10	Helmholtz Association (Germany)	34	2018
11	State University System of Florida (the United States)	34	2018
12	Jefferson University (the United States)	32	2018
13	University of Florida (the United States)	32	2018
14	Mayo Clinic (the United States)	30	2017
15	Stanford University (the United States)	30	2018
16	German Cancer Research Center (DKFZ) (Germany)	30	2018
17	University System of Ohio (the United States)	25	2014
18	Northwestern University (the United States)	25	2017
19	University of Freiburg (Germany)	24	2018
20	University of Texas Health Science Center Houston (the United States)	23	2014

### References

The analysis of reference co-citations was executed utilizing CiteSpace software. A total of 86 references exhibited more than 10 co-citations, while a subset of 48 references amassed co-citations exceeding 20 occurrences (Table S2, Supplemental Digital Content 1, http://links.lww.com/JS9/B930). Among the 86 references with co-citations of over 10 times, there were 13 references published in 2017 and 2018, and 11 references published in 2014, 2019, and 2020. And it showed the fast development of TTFields in these years (especially after 2017). The 10 most highly cited and co-cited references within the field of TTFields are delineated in Tables 2 and 3 (encompassing their core contents or results), respectively. In Table 2, there are three references associated with clinical trials, and two references are associated with in vitro studies. And other references are associated with reviews on glioma/glioblastoma and meningiomas, or TTFields. The references with the highest citation numbers are the clinical trial (NCT00916409) of Stupp R *et al*.^3,27^ (*n*=1200 and *n*=785), which proved the stronger antitumor effect of the combination therapy (TTFields plus Temozolomide) compared to single Temozolomide in patients with newly diagnosed GBM. And it is followed by reviews of Tan AC *et al*.^28^ (*n*=722), Weller M *et al*.^29^ (*n*=667), and Davis ME^30^ (*n*=640), which are associated with management of glioma/glioblastoma.

**Table 2:** The top 10 references with the highest citations in this field from WoS platform.

No.	Title	Journal	First Author	Citation	Year	Core contents or results
1	Effect of Tumor-Treating Fields Plus Maintenance Temozolomide vs Maintenance Temozolomide Alone on Survival in Patients With Glioblastoma A Randomized Clinical Trial	JAMA	Stupp R *et al.* ^3^	1200	2017	The final analysis of NCT00916409. Among 695 patients with GBM (after initial radiochemotherapy), mPFS and mOS were 6.7 versus 4.0 months and 20.9 versus 16.0 months in the TTFields plus TMZ group and TMZ-alone group, respectively
2	Maintenance Therapy With Tumor-Treating Fields Plus Temozolomide vs Temozolomide Alone for Glioblastoma A Randomized Clinical Trial	JAMA	Stupp R *et al.* ^27^	785	2015	The interim analysis of NCT00916409. Among 315 patients with GBM (after initial radiochemotherapy), mPFS and mOS were 7.1 versus 4.0 months and 20.5 versus 15.6 months in the TTFields plus TMZ group and TMZ-alone group, respectively
3	Management of glioblastoma: State of the art and future directions	CA Cancer J Clin	Tan AC *et al.* ^28^	722	2020	It reviewed the status and direction of GBM management, stressing the importance of multimodality (including TTFields) approaches and biomarker-enrichment strategies
4	European Association for Neuro-Oncology (EANO) guideline on the diagnosis and treatment of adult astrocytic and oligodendroglial gliomas	Lancet Oncol	Weller M *et al.* ^29^	667	2017	The European Association for Neuro-Oncology guideline of 2017
5	Glioblastoma: Overview of Disease and Treatment	Clin J Oncol Nurs	Davis ME *et al.* ^30^	640	2016	A brief review of treatment options (including TTFields) for GBM
6	Alternating electric fields arrest cell proliferation in animal tumor models and human brain tumors	Proc Natl Acad Sci U S A	Kirson ED *et al.* ^31^	539	2007	A preclinical study of alternating electric fields influencing tumor cell proliferation, and a pilot clinical trial of TTFields in 10 patients with recurrent GBM
7	Glioma Subclassifications and Their Clinical Significance	Neurotherapeutics	Chen R *et al.* ^32^	384	2017	It reviewed various subclassifications of gliomas, along with their clinical significance
8	An overview of meningiomas	Future Oncol	Buerki RA *et al.* ^33^	203	2018	An overview of meningiomas, including its exploring trial of TTFields
9	Mitotic Spindle Disruption by Alternating Electric Fields Leads to Improper Chromosome Segregation and Mitotic Catastrophe in Cancer Cells	Sci Rep	Giladi M *et al.* ^34^	179	2015	TTFields can decrease the ratio between polymerized and total tubulin, and prevent proper mitotic spindle assembly
10	Tumor-Treating Fields: A Fourth Modality in Cancer Treatment	Clin Cancer Res	Mun EJ *et al.* ^1^	173	2018	Review of TTFields in many cancers including GBM, pancreatic cancer, ovarian cancer, lung cancer, malignant mesothelioma

mOS, median overall survival; mPFS, median progression-free survival; TMZ, temozolomide; TTFields, Tumor treating fields.

**Table 3:** The top 10 references with the highest co-citations in this field from citespace.

No.	First author	Title	Journal	Year	Co-citation	Centrality	Core contents or results
1	Stupp R *et al.* ^3^	Effect of Tumor-Treating Fields Plus Maintenance Temozolomide vs Maintenance Temozolomide Alone on Survival in Patients With Glioblastoma: A Randomized Clinical Trial	JAMA	2017	186	0.01	The final analysis of NCT00916409. Among 695 patients with GBM (after initial radiochemotherapy), mPFS and mOS were 6.7 versus 4.0 months and 20.9 versus 16.0 months in the TTFields plus TMZ group and TMZ-alone group, respectively
2	Stupp R *et al.* ^27^	Maintenance Therapy With Tumor-Treating Fields Plus Temozolomide vs Temozolomide Alone for Glioblastoma: A Randomized Clinical Trial	JAMA	2015	100	0.06	The interim analysis of NCT00916409. Among 315 patients with GBM (after initial radiochemotherapy), mPFS and mOS were 7.1 versus 4.0 months and 20.5 versus 15.6 months in the TTFields plus TMZ group and TMZ-alone group, respectively
3	Mun EJ *et al.* ^1^	Tumor-Treating Fields: A Fourth Modality in Cancer Treatment	CLIN CANCER RES	2018	64	0.01	Review of TTFields in many cancers including GBM, pancreatic cancer, ovarian cancer, lung cancer, malignant mesothelioma
4	Giladi M *et al.* ^34^	Mitotic Spindle Disruption by Alternating Electric Fields Leads to Improper Chromosome Segregation and Mitotic Catastrophe in Cancer Cells	SCI REP	2015	59	0.02	TTFields can decrease the ratio between polymerized and total tubulin, and prevent proper mitotic spindle assembly
5	Gera N *et al.* ^35^	Tumor treating fields perturb the localization of septins and cause aberrant mitotic exit	PLOS ONE	2015	56	0.01	TTFields can affect cancer cell division by interfering with cytokinetic cleavage furrow (CCF) function and that at least one key protein, Septin
6	Toms SA *et al.* ^36^	Increased compliance with tumor treating fields therapy is prognostic for improved survival in the treatment of glioblastoma: a subgroup analysis of the EF-14 phase III trial	J NEURO-ONCOL	2019	55	0	A subgroup analysis of EF-14 trial, showing that increased compliance with TTFields is prognostic for improved survival in GBM treatment
7	Karanam NK *et al.* ^37^	Tumor-treating fields elicit a conditional vulnerability to ionizing radiation via the downregulation of BRCA1 signaling and reduced DNA double-strand break repair capacity in nonsmall cell lung cancer cell lines	CELL DEATH DIS	2017	54	0.04	Molecular mechanisms and ionizing radiation of TTFields in nonsmall cell lung cancer cell lines, with the stress of the combined TTFields and radiotherapy
8	Taphoorn MJB *et al.* ^38^	Influence of Treatment With Tumor-Treating Fields on Health-Related Quality of Life of Patients With Newly Diagnosed Glioblastoma: A Secondary Analysis of a Randomized Clinical Trial	JAMA ONCOL	2018	54	0.02	A secondary analysis of EF-14 trial, showing that health-related quality of life did not differ significantly between treatment arms except for itchy skin
9	Giladi M *et al.* ^39^	Tumor treating fields (TTFields) delay DNA damage repair following radiation treatment of glioma cells	RADIAT ONCOL	2017	51	0.01	TTFields can delay DNA damage repair after radiotherapy, with the stress of the combined TTFields and radiotherapy
10	Stupp R *et al.* ^2^	NovoTTF-100A versus physician’s choice chemotherapy in recurrent glioblastoma: a randomised phase III trial of a novel treatment modality	EUR J CANCER	2012	50	0.03	No improved mOS was demonstrated in single TTFields therapy, compared with single chemotherapy

mOS, median overall survival; mPFS, median progression-free survival; TMZ, temozolomide; TTFields, Tumor treating fields.

Within the cadre of the top 10 co-cited references delineated in Table 3, five are oriented towards clinical trials, four constitute original articles, and one is a review. This distribution underscores the confluence of preclinical research and clinical investigation in shaping the scientific discourse in the field of TTFields. The top two co-cited references are the same as the top two cited references. Besides, in another highly co-cited reference (a phase III clinical trial published in 2012), Stupp *et al*.^2^ compared NovoTTF-100A with chemotherapy in patients with recurrent glioblastoma. The other two references were the further analyses of the clinical trial NCT00916409. One proved that the increased compliance with TTFields is prognostic for improved survival, and the other tested the influence of TTFields on health-related quality of life of patients with newly diagnosed glioblastoma. Besides, In 2018 Mun *et al*.^1^ reviewed the current status of TTFields, and described it as a fourth modality of cancer treatment. The other four original articles were exploring the antitumor mechanism of TTFields, mainly concentrated on mitosis and DNA damage repair.

### Keywords

#### General analysis of keywords

Utilizing Citespace for comprehensive keyword analysis, our study encompassed 515 keywords interconnected through 1968 relationships, yielding a density of 0.0149. Initial data procurement involved determining the frequencies and betweenness centralities of all keywords under consideration. Subsequent to these general evaluations, the keywords were compartmentalized into three categorical delineations: diseases (Table 4), treatments/therapies (Table 5), and miscellaneous keywords (Table 6). In Table 4, it is found that all disease-related keywords were cancers/tumors. Glioblastoma was the keyword with the highest frequency (*n*=88), followed by cancer (*n*=71). Recurrent glioblastoma (*n*=64) and newly diagnosed glioblastoma (*n*=60) were other two high-frequency keywords. And a series of keywords were related to gliomas, including malignant glioma (*n*=25), glioma (*n*=16), glioblastoma multiforme (*n*=13), high grade glioma (*n*=8), etc. Besides gliomas of various types or classifications, lung cancer was identified as another cancer utilizing TTFields as a treatment approach, based on three keywords including cell lung cancer (*n*=5), lung cancer (*n*=4), and nonsmall cell lung cancer (*n*=2). Other types of cancers/tumors included MPM (*n*=3), brain metastases (*n*=3), ovarian cancer (*n*=2), triple-negative breast cancer (*n*=2), and pancreatic ductal adenocarcinoma (*n*=2).

**Table 4:** Frequency and centrality of keywords associated with diseases.

No.	Frequency	Centrality	Appearance year	Keyword
1	88	0.26	2011	glioblastoma
2	71	0.07	2008	cancer
3	64	0	2012	recurrent glioblastoma
4	60	0.12	2015	newly diagnosed glioblastoma
5	34	0.39	2007	malignant glioma
6	25	0.05	2014	brain tumor
7	16	0.07	2012	glioma
8	13	0.26	2007	glioblastoma multiforme
9	8	0.01	2019	high grade glioma
10	5	0.01	2011	cell lung cancer
11	4	0.04	2015	lung cancer
12	3	0.03	2016	breast cancer
13	3	0	2015	malignant pleural mesothelioma
14	3	0	2015	brain metastases
15	2	0.06	2017	low grade gliomas
16	2	0.05	2011	nonsmall cell lung cancer
17	2	0.03	2016	ovarian cancer
18	2	0	2023	triple-negative breast cancer (tnbc)
19	2	0	2018	progressive glioblastoma
20	2	0	2020	nervous system tumors
21	2	0	2017	anaplastic oligodendroglioma
22	2	0	2021	pancreatic ductal adenocarcinoma

**Table 5:** Frequency and centrality of keywords associated with treatments/therapies.

No.	Frequency	Centrality	Appearance year	Keyword
1	302	0.04	2007	tumor treating fields
2	110	0.01	2007	temozolomide
3	91	0.09	2012	radiotherapy (radiation therapy)
4	61	0.08	2012	adjuvant temozolomide
5	59	0.05	2013	novottf-100a
6	52	0.02	2011	chemotherapy
7	43	0.06	2011	alternating electric fields
8	38	0.32	2008	electric fields
9	36	0	2015	bevacizumab
10	17	0.04	2014	physicians choice chemotherapy
11	10	0.1	2015	electromagnetic fields
12	8	0	2017	drug delivery
13	7	0.01	2017	lomustine
14	7	0	2019	maintenance temozolomide
15	6	0.08	2014	single agent bevacizumab
16	6	0.01	2014	dielectric property
17	5	0.02	2016	combination therapy
18	4	0	2017	targeted therapy
19	4	0.02	2016	immunotherapy
20	3	0	2022	rindopepimut

**Table 6:** Frequency and centrality of other keywords.

No.	Frequency	Centrality	Appearance year	Keyword
1	65	0.06	2014	survival
2	57	0.06	2013	trial
3	55	0	2013	randomized phase iii
4	50	0.02	2012	brain
5	38	0.06	2015	in vitro
6	36	0.04	2017	central nervous system
7	35	0.04	2014	disruption
8	25	0.15	2013	phase iii
9	24	0.01	2018	cell proliferation
10	24	0.02	2013	concomitant
11	23	0.18	2007	phase ii
12	22	0.08	2014	proliferation
13	19	0.05	2015	open label
14	15	0.04	2017	blood–brain barrier
15	12	0	2017	expression
16	10	0	2016	clinical trial
17	8	0.01	2019	modality
18	7	0.02	2014	tissue
19	7	0	2019	tumor microenvironment
20	7	0.03	2019	t cells

In Table 5, lots of treatment approaches were identified, which were centered in the keyword of TTfileds (*n*=302). Besides, many keywords that were associated with TTfileds had also been recognized, including novottf-100a (*n*=59), alternating electric fields (*n*=43), electric fields (*n*=38), electromagnetic fields (*n*=10), and dielectric property (*n*=6). Other treatment approaches in this table are probably TTfileds-based combined therapeutic approaches, or TTfileds-compared approaches. The keywords of these treatment approaches or drugs included temozolomide (*n*=110), radiotherapy (radiation therapy) (*n*=91), adjuvant temozolomide (*n*=61), chemotherapy (*n*=52), bevacizumab (*n*=36), physicians choice chemotherapy (*n*=17), drug delivery (*n*=8), lomustine (*n*=7), maintenance temozolomide (*n*=7), single agent bevacizumab (*n*=6), etc.

Table 6 introduced some other high-frequency keywords. Among these keywords, ‘clinical trial’-related keywords including trial (*n*=57), randomized phase iii (*n*=55), phase iii (*n*=25), phase ii (*n*=23), open label (*n*=19), and clinical trial (*n*=10). And this indicted the active attempts of TTfileds in the stage of clinical trials in recent years. Oher high-frequency keywords included survival (*n*=65), in vitro (*n*=38), cell proliferation (*n*=24)/proliferation (*n*=22), blood–brain barrier (*n*=15), tumor microenvironment (*n*=7), and t cells (*n*=7).

#### The clustering analysis of keywords

**Figure 4:**
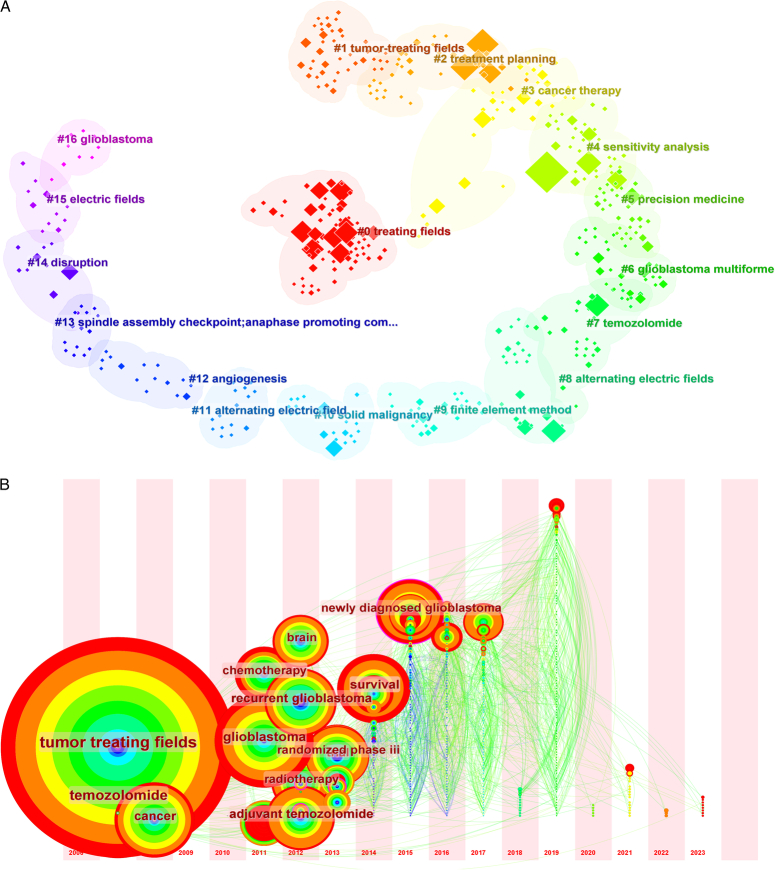
(A) Citespace-generated clustering map of keywords with modularity Q=0.8054, weighted mean silhouette S=0.9133, and harmonic mean (Q, S)=0.8559. (B) Timezone analysis of keyword emergence and prevalence.

The clustering of keywords into 17 distinct categories provides specific focal points that capture the array of research topics currently under investigation in the field of TTFields (Fig. 4A). For instance, Cluster #0, designated as ‘treating fields’, encapsulates foundational research on the modality itself, whereas Cluster #1, labeled “tumor-treating fields,” likely delves into the application and effectiveness of TTFields in various tumor types. Cluster #6, focused on ‘glioblastoma multiforme’, indicates an evident concentration on this particular cancer subtype, possibly revealing a significant emphasis on TTFields in its treatment paradigm. Similarly, the presence of clusters like #7 ‘temozolomide’ and #12 ‘angiogenesis’ suggests that combinatorial or synergistic treatments may be a subject of research enthusiasm. Cluster #8 and #11, both dealing with ‘alternating electric fields’, point toward a scientific commitment to understanding the biophysical mechanisms behind TTFields. Inclusion of clusters like #5 ‘precision medicine’ hints at a more personalized approach to using TTFields, possibly exploring genetic or molecular markers to predict therapy success.

By providing these well-defined thematic clusters, the analysis furnishes a structured outline of the research landscape, enabling scholars and clinicians to grasp the array of intellectual threads that make up the fabric of this rapidly evolving field. This organization into discrete clusters will likely prove indispensable for future research, by identifying both areas of current research strength and potential gaps warranting exploration.

#### The timeline view, timezone view, and landscape of keywords

**Figure 5:**
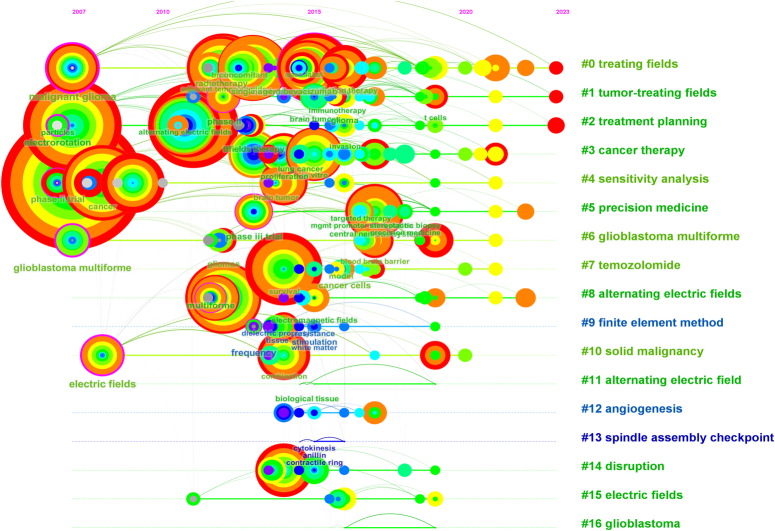
Temporal analysis of keyword clusters, highlighting longitudinal trends, and pivotal milestones.

The timeline view analysis furnishes an insightful perspective on the evolution of the scientific dialog surrounding TTFields (Fig. 5). Notably, clusters such as #0 ‘treating fields’, #2 ‘treatment planning’, and #6 ‘glioblastoma multiforme’ exhibit a longitudinal vitality, indicating their sustained relevance in the research community over an extended period. Moreover, the prospective longevity of clusters like #1 ‘tumor-treating fields’, #5 ‘precision medicine’, and #8 ‘alternating electric fields’ underscores their emerging or enduring significance in the field.

In contrast to the timeline view, the timezone view analysis provides a different layer of understanding (Fig. 4B). By anchoring each keyword’s node to its year of first appearance, this approach reveals that high-frequency keywords are predominantly clustered before 2017. This observation could imply either foundational work primarily conducted during this period or a degree of saturation in certain research themes. However, the subsequent burst of novel keywords in 2019 suggests a reinvigoration of the field, potentially signaling the advent of fresh paradigms or innovative approaches to TTFields.

**Figure 6:**
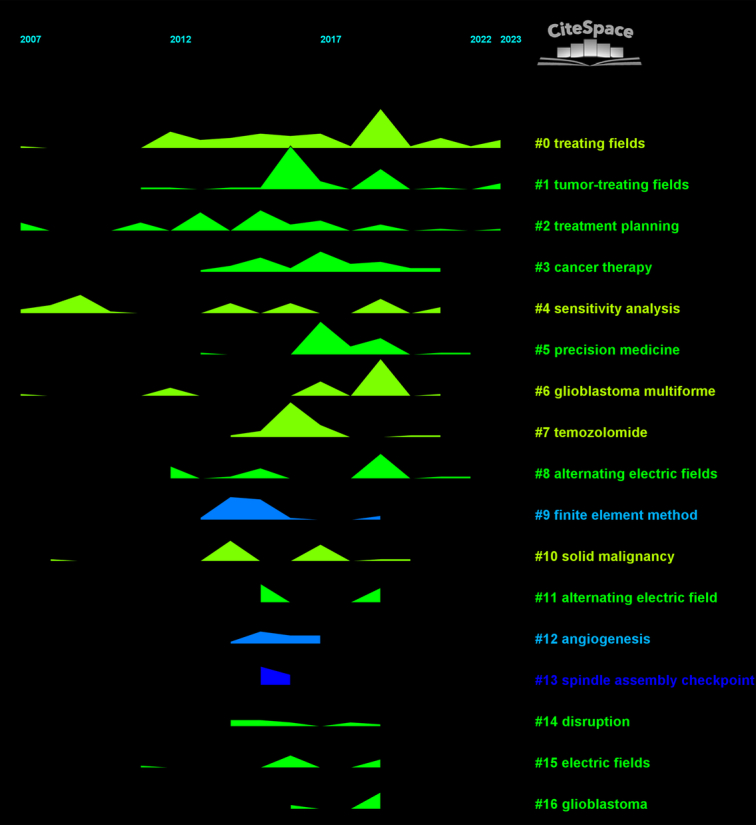
Multidimensional scaling plot delineating the landscape of keyword clusters, with color-coded density or frequency markers.

The landscape analysis further enriches our understanding by capturing the key milestones and breakthroughs for each cluster (Fig. 6). Concentration of progress between 2015 and 2019 corroborates the timezone view and may indicate a period of consolidation, following which novel research avenues have begun to proliferate. This consistency across timeline, timezone, and landscape views collectively furnishes a nuanced panorama of the TTFields research sphere, providing researchers, clinicians, and policymakers a comprehensive understanding of past achievements, current trends, and future directions.

## Discussion

This scientometric analysis provides a robust, comprehensive view of the burgeoning field of TTFields, offering both quantitative and qualitative insights. The surge in research output after 2016 is a testament to the growing academic and clinical interest in TTFields. This crescendo reached a pinnacle in 2019, a year that seems to be a nexus for both novel keyword emergence and research output, suggesting not just quantitative growth but also the potential advent of paradigm shifts or new methodological approaches in the field. The geographical distribution of research output delineates a clear hierarchy, with the United States, Israel, Germany, and Switzerland at the forefront. This global stratification extends to institutional contributions, with a preponderance of American and Israeli institutions dominating the field—Novocure being notably preeminent. Such geographical and institutional trends, aside from illustrating the centers of academic gravity, also hint at potential regional foci in TTFields application or research themes. Subject category analysis further reveals that TTFields is an interdisciplinary area, mainly straddling Oncology, Clinical Neurology, and Radiology, Nuclear Medicine and Medical Imaging. This corroborates the complex and multifaceted nature of TTFields technology, necessitating expertise in multiple domains. In terms of individual contributions, authors such as Weinberg Uri, Giladi Moshe, and Palti Yoram have surfaced as significant influencers. Their copious output, in terms of both quantity and citations, indicates their foundational roles in the intellectual architecture of this field. This observation is further accentuated by the co-citation analysis, which emphasizes the imprint of these thought leaders on the collective research consciousness. Journal-level analysis is consistent with the interdisciplinary nature of the TTFields research ecosystem. Journals such as ‘Neuro-Oncology’, ‘Cancer Research’, and ‘International Journal of Radiation Oncology Biology Physics’ serve as key platforms for disseminating TTFields-related studies. Their counterpart journals in terms of citations, including ‘Clinical Journal of Oncology Nursing’, ‘Cancers’, and ‘Journal of Neuro-Oncology’, underscore the widespread clinical implications of TTFields, particularly in the realm of neuro-oncology. The reference and keyword analyses, intended to be the fulcrum of this article, will necessarily incorporate and build upon these foundational elements. By parsing the core references and key thematic clusters, this work aims to furnish a nuanced, multidimensional understanding of TTFields, tracing its evolutionary trajectory and offering prospective directions for researchers and clinicians alike.

### Mechanisms of TTFields

In the analyses of references, there were five articles exploring the mechanism of TTFields for cancers/tumors^31–35^. Moreover, several clustering keywords such as alternating electric fields, angiogenesis, spindle assembly checkpoint, disruption also indicted the wide exploration of mechanisms of TTFields in the past decade. In recent years, there have been dozens of both research and review articles that well clarified the antitumor mechanisms of TTFields^14–16,36–39^. Thus, our discussion would like to simply summarize these antitumor mechanisms or strategies: 1) disruption of cell cycle and mitosis; 2) interruption of DNA damage response; 3) disruption of cellular components (cell membrane, organelle, nuclear structure, or cytoskeleton); 4) change of ion channels; 5) interference of cell metabolism; 6) induction of cell death (cell autophagy, apoptosis, pyrotosis, necrosis, necroptosis, or immunogenetic death); 7) inhibition of tumor invasion, migration and metastasis; 8) inhibition of tumor angiogenesis; 9) activation of immunosuppressive tumor microenvironment; 10) enhancement of blood–brain barrier (BBB) penetration.

Besides, TTFields therapy shows diverse efficacy under different electric intensities and frequencies and is influenced by the conductivity of the skull, tumor position, and tissue homogeneity. The tenth mechanism or strategy is of great use to BBB crossing-based therapies such as chemotherapeutic or immunotherapeutic drugs. And it also lays the foundation of TTfileds combined with nano-based therapies^40–42^. Moreover, since synergistic antitumor effects can be induced by different treatment approaches (either same or different antitumor mechanisms), exploration of antitumor mechanisms of TTFields, especially TTFields combined with other therapies, should be kept up in the future^15,43^.

### Application of TTFields

Currently, TTFields have been approved by FDA for treating patients with glioblastoma (both recurrent and newly diagnosed) and MPM^44^. As scientometric analysis showed, research on TTFields mainly focuses on cancer treatment, especially the treatment of glioblastoma (both recurrent and newly diagnosed) or malignant glioma. And several meta-analyses had well proved the enhanced efficacy of TTFields therapy plus standard of care compared with standard of care^45–48^. As the other approved indications, MPM has also been studied in this field. Besides, TTFields have also been studied for the treatment of lung cancer especially nonsmall cell lung cancer (NSCLC), brain metastases of malignant tumors, ovarian cancer, breast cancer, pancreatic cancer, etc. Moreover, the completed clinical trials of TTFields were obtained from www.clinicaltrials.gov (Table 7). While Table 7 exhibits similar pattern of TTFields’ applications to scientometric analysis, it was found that TTFields had also been used for combating with COVID-19^49^. Furthermore, the ongoing clinical trials of TTFields were obtained (Table S3, Supplemental Digital Content 1, http://links.lww.com/JS9/B930). Table S3 (Supplemental Digital Content 1, http://links.lww.com/JS9/B930) indicted the active status of TTFields used for treatment of various types of cancers including brain tumors (high grade glioma especially glioblastoma, ependymoma, atypical and anaplastic meningioma, metastatic brain cancer), NSCLC, metastatic lung cancer, hepatic cancer, metastatic hepatic cancer, pancreatic cancer, metastatic pancreatic cancer, melanoma metastasis, ovarian cancer, etc.

**Table 7:** The completed clinical trials of TTfields from www.clinicaltrials.gov.

NCT number	Phase	Study title	Cancer types	Interventions
NCT00749346	I/II	NovoTTF-100L in Combination With Pemetrexed (Alimta®) for Advanced Nonsmall Cell Lung Cancer	Advanced NSCLC	TTfields plus Pemetrexed
NCT02893137	I	Enhancing Optune Therapy With Targeted Craniectomy	Recurrent Glioblastoma	TTfields plus Craniectomy
NCT01894061	II	NovoTTF-100A With Bevacizumab (Avastin) in Patients With Recurrent Glioblastoma	Recurrent Glioblastoma	TTfields plus Bevacizumab
NCT00916409	III	Effect of NovoTTF-100A Together With Temozolomide in Newly Diagnosed Glioblastoma Multiforme (GBM)	Newly Diagnosed Glioblastoma	TTfields plus Temozolomide
NCT04953234	Not applicable	Effect of Tumor Treating Fields (TTFields, 150 kHz) Concomitant With Best Standard of Care for the Treatment of Hospitalized COVID-19 Patients and Continued Treatment Following Discharge	COVID-19	TTfields
NCT03501134	Observational	Quality of Life of Patients With Glioblastoma (GBM) Treated With Tumor-Treating Fields	Glioblastoma	TTfields
NCT03232424	I	NovoTTF-200A and Temozolomide Chemoradiation for Newly Diagnosed Glioblastoma	Newly Diagnosed Glioblastoma	TTfields plus Temozolomide
NCT02397928	II	Safety and Efficacy of TTFields (150 kHz) Concomitant With Pemetrexed and Cisplatin or Carboplatin in Malignant Pleural Mesothelioma (STELLAR)	Malignant Pleural Mesothelioma	TTfields plus Pemetrexed and Cisplatin (or Carboplatin)
NCT00379470	III	Effect of NovoTTF-100A in Recurrent Glioblastoma Multiforme (GBM)	Recurrent Glioblastoma	TTfields
NCT02903069	I	Study of Marizomib With Temozolomide and Radiotherapy in Patients With Newly Diagnosed Brain Cancer	Newly Diagnosed Glioblastoma	TTfields plus Marizomib and Temozolomide and Radiotherapy

It is acknowledged that TTFields have well served as an assistant role instead of a dominant approach in cancer treatment. The scientometric analysis also showed that temozolomide (the standard chemotherapeutic drug for glioma/glioblastoma treatment) was not only a high-frequency keyword but also a clustering keyword. Radiotherapy, which is an essential part of the treatment of many cancers including glioma/glioblastoma, was also on the list of high-frequency keywords. Another high-frequency keyword Bevacizumab is a crucial drug for patients with recurrent glioblastoma^50,51^, or MPM^52^. Table 7 and Table S3 (Supplemental Digital Content 1, http://links.lww.com/JS9/B930) also supported the main applications of TTFields focus on the combination of TTFields and other therapies. More and more studies are exploring the feasibility and efficacy of TTFields combined with other therapies, either standard care procedures or novel promising therapies^15,53,54^. For the treatment of glioblastoma or brain tumors, some special strategies have also been explored and applied, such as skull modulated strategies^55,56^, and enhanced BBB penetration for drug delivery^15^. Besides, it is found that TTFields are preferred to be used in the treatment of advanced, recurrent, or metastatic cancers, indicating its important goal and purpose.

## Conclusion

This scientometric analysis of TTFields has well demonstrated the current research status and hot topics of TTFields. In recent years, publication numbers have been stable at high values, and citation numbers have been increasing greatly. The United States and Israel were the top two countries with the highest publication numbers, followed by Germany and Switzerland. Scientometric analyses of keywords indicated that clinical applications and antitumor mechanisms are probably the two main parts of current research on TTFields. Most clinical trials of TTFields focus on the treatment of glioblastoma. And a variety of other cancers such as lung cancer especially nonsmall cell lung cancer, hepatic cancer, other brain tumors, etc. have also been studied in both clinical trials and preclinical studies. Overall, TTFields therapy is a promising therapeutic approach for cancers, mainly serving as an assistant role to combine with other feasible therapies. The future studies of TTFields may probably focus on the explorations of antitumor mechanisms and clinical applications of TTFields therapy combined with other therapies. And more approved indications of TTFields therapy is expected.

## Limitations

There are several limitations associated with this scientometric analysis that need to be considered in the context of the methods employed. Firstly, there is a potential of English language bias in the database used, which may result in the omission of many relevant non-English articles. Secondly, relying on a single database restricts the ability to identify all relevant publications, as it may not cover the entire spectrum of relevant literature. Thirdly, limiting the search to specific fields such as title, abstract, and keywords could potentially exclude relevant publications from being identified during the search. However, conducting a search across all fields is likely to retrieve a substantial number of irrelevant publications. Lastly, it is important to note that these limitations may be mitigated or resolved through improvements of retrieval databases and relevant software tools.

## Ethical approval

Not applicable.

## Consent

Not applicable.

## Sources of funding

This work was supported by the Natural Science Foundation of Jilin Province, China (Grant No. 20200201491JC), and the Health Planning Commission of Jilin Province, China (Grant No. 2017J046).

## Author contribution

Y.X., F.Y., J.C., and X.H.: conceived the study and performed critical revision of the manuscript; Y.X., F.Y., and J.C.: designed the study, performed statistical analyses, and drafted the manuscript, and wrote the manuscript; Y.X. and F.Y.: performed the article retrieval and data interpretation. All authors read and approved the final manuscript.

## Conflicts of interest disclosure

The authors declare that they have no financial conflicts of interest with regard to the content of this report.

## Research registration unique identifying number (UIN)

1. Name of the registry: not applicable.
2. Unique identifying number or registration ID: not applicable.
3. Hyperlink to your specific registration (must be publicly accessible and will be checked): not applicable.

## Guarantor

Professor Xinyu Hong.

## Data availability statement

All the data could be contact with the corresponding author Professor. Xinyu Hong (hongxy@jlu.edu.cn) with scientific purpose.

## Provenance and peer review

The paper is not invited.

## Assistance with the study

Not applicable.

## Presentation

Not applicable.

## Supplementary Material
