# Supplementary material for: Advancements and current trends in tumor treating fields: a scientometric analysis
Source: Int J Surg. 2024 Feb 13;110(5):2978–91. doi: 10.1097/JS9.0000000000001151 (PMC11093503; doi:10.1097/JS9.0000000000001151)
Supplement: Supplementary file 1 [file js9-110-2978-s001.docx]

**Supplementary Materials**

**Table S1** The publications and betweenness centralities of countries.

| Country | Start year | Publication | Betweenness centrality |
| --- | --- | --- | --- |
| USA | 2012 | 540 | 0.47 |
| ISRAEL | 2006 | 422 | 0.49 |
| GERMANY | 2016 | 274 | 0.08 |
| SWITZERLAND | 2006 | 173 | 0.08 |
| PEOPLES R CHINA | 2018 | 78 | 0.16 |
| SPAIN | 2015 | 57 | 0.24 |
| ITALY | 2012 | 57 | 0.95 |
| SOUTH KOREA | 2015 | 44 | 0.01 |
| FRANCE | 2007 | 37 | 1.01 |
| CANADA | 2015 | 35 | 0.39 |
| CZECH REPUBLIC | 2006 | 32 | 0.67 |
| ENGLAND | 2016 | 29 | 0.08 |
| BELGIUM | 2016 | 24 | 0.04 |
| PORTUGAL | 2014 | 23 | 0 |
| JAPAN | 2016 | 21 | 0 |
| DENMARK | 2016 | 20 | 0.22 |
| NETHERLANDS | 2017 | 20 | 0.27 |
| POLAND | 2016 | 17 | 0.3 |
| AUSTRIA | 2017 | 12 | 0.02 |
| SWEDEN | 2012 | 10 | 0.03 |
| AUSTRALIA | 2018 | 9 | 0.1 |
| SOUTH AFRICA | 2018 | 5 | 0 |
| TAIWAN | 2018 | 4 | 0 |
| TURKEY | 2020 | 3 | 0 |
| EGYPT | 2020 | 3 | 0 |
| PAKISTAN | 2018 | 3 | 0.04 |
| BRAZIL | 2022 | 3 | 0.18 |
| IRAN | 2021 | 3 | 0.24 |
| INDIA | 2021 | 3 | 0 |
| IRELAND | 2018 | 2 | 0 |
| RUSSIA | 2018 | 2 | 0 |
| HUNGARY | 2019 | 2 | 0 |
| SLOVENIA | 2021 | 1 | 0 |
| TRINIDAD TOBAGO | 2021 | 1 | 0 |
| U ARAB EMIRATES | 2021 | 1 | 0 |
| SERBIA | 2023 | 1 | 0 |
| GREECE | 2018 | 1 | 0 |
| QATAR | 2021 | 1 | 0 |
| BULGARIA | 2023 | 1 | 0 |
| MALAYSIA | 2022 | 1 | 0 |
| NORWAY | 2021 | 1 | 0 |
| SINGAPORE | 2020 | 1 | 0 |
| BANGLADESH | 2023 | 1 | 0.04 |
| CROATIA | 2023 | 1 | 0 |
| SCOTLAND | 2020 | 1 | 0 |
| SAUDI ARABIA | 2022 | 1 | 0 |
| BELGIUM | 2012 | 540 | 0.47 |

**Table S2** The co-citations of publications relevant to this field.

| No. | Year | Brief introduction | Counts | Betweenness centrality |
| --- | --- | --- | --- | --- |
|  | 2017 | Stupp R, 2017, JAMA-J AM MED ASSOC, V318, P2306, DOI 10.1001/jama.2017.18718 | 186 | 0.01 |
|  | 2015 | Stupp R, 2015, JAMA-J AM MED ASSOC, V314, P2535, DOI 10.1001/jama.2015.16669 | 100 | 0.06 |
|  | 2018 | Mun EJ, 2018, CLIN CANCER RES, V24, P266, DOI 10.1158/1078-0432.CCR-17-1117 | 64 | 0.01 |
|  | 2015 | Giladi M, 2015, SCI REP-UK, V5, P0, DOI 10.1038/srep18046 | 59 | 0.02 |
|  | 2015 | Gera N, 2015, PLOS ONE, V10, P0, DOI 10.1371/journal.pone.0125269 | 56 | 0.01 |
|  | 2019 | Toms SA, 2019, J NEURO-ONCOL, V141, P467, DOI 10.1007/s11060-018-03057-z | 55 | 0 |
|  | 2017 | Karanam NK, 2017, CELL DEATH DIS, V8, P0, DOI 10.1038/cddis.2017.136 | 54 | 0.04 |
|  | 2018 | Taphoorn MJB, 2018, JAMA ONCOL, V4, P495, DOI 10.1001/jamaoncol.2017.5082 | 54 | 0.02 |
|  | 2017 | Giladi M, 2017, RADIAT ONCOL, V12, P0, DOI 10.1186/s13014-017-0941-6 | 51 | 0.01 |
|  | 2012 | Stupp R, 2012, EUR J CANCER, V48, P2192, DOI 10.1016/j.ejca.2012.04.011 | 50 | 0.03 |
|  | 2017 | Silginer M, 2017, CELL DEATH DIS, V8, P0, DOI 10.1038/cddis.2017.171 | 46 | 0.01 |
|  | 2018 | Chang E, 2018, CELL DEATH DISCOV, V4, P0, DOI 10.1038/s41420-018-0130-x | 43 | 0.01 |
|  | 2016 | Louis DN, 2016, ACTA NEUROPATHOL, V131, P803, DOI 10.1007/s00401-016-1545-1 | 40 | 0.1 |
|  | 2018 | Shteingauz A, 2018, CELL DEATH DIS, V9, P0, DOI 10.1038/s41419-018-1085-9 | 40 | 0.01 |
|  | 2019 | Ballo MT, 2019, INT J RADIAT ONCOL, V104, P1106, DOI 10.1016/j.ijrobp.2019.04.008 | 39 | 0.02 |
|  | 2016 | Hottinger AF, 2016, NEURO-ONCOLOGY, V18, P1338, DOI 10.1093/neuonc/now182 | 39 | 0.01 |
|  | 2019 | Ceresoli GL, 2019, LANCET ONCOL, V20, P1702, DOI 10.1016/S1470-2045(19)30532-7 | 38 | 0.01 |
|  | 2020 | Karanam NK, 2020, TRANSL RES, V217, P33, DOI 10.1016/j.trsl.2019.10.003 | 35 | 0.01 |
|  | 2021 | Rominiyi O, 2021, BRIT J CANCER, V124, P697, DOI 10.1038/s41416-020-01136-5 | 34 | 0.04 |
|  | 2019 | Rivera F, 2019, PANCREATOLOGY, V19, P64, DOI 10.1016/j.pan.2018.10.004 | 34 | 0.02 |
|  | 2018 | Vergote I, 2018, GYNECOL ONCOL, V150, P471, DOI 10.1016/j.ygyno.2018.07.018 | 34 | 0.02 |
|  | 2020 | Voloshin T, 2020, CANCER IMMUNOL IMMUN, V69, P1191, DOI 10.1007/s00262-020-02534-7 | 32 | 0.01 |
|  | 2014 | Chinot OL, 2014, NEW ENGL J MED, V370, P709, DOI 10.1056/NEJMoa1308345 | 30 | 0.03 |
|  | 2018 | Wenger Cornelia, 2018, IEEE REV BIOMED ENG, V11, P195, DOI 10.1109/RBME.2017.2765282 | 30 | 0.01 |
|  | 2017 | Perry JR, 2017, NEW ENGL J MED, V376, P1027, DOI 10.1056/NEJMoa1611977 | 29 | 0.02 |
|  | 2014 | Lacouture Mario E, 2014, SEMIN ONCOL, V41 Suppl 4, PS1, DOI 10.1053/j.seminoncol.2014.03.011 | 28 | 0 |
|  | 2018 | Kessler AF, 2018, CELL DEATH DISCOV, V4, P0, DOI 10.1038/s41420-018-0079-9 | 28 | 0 |
|  | 2014 | Gilbert MR, 2014, NEW ENGL J MED, V370, P699, DOI 10.1056/NEJMoa1308573 | 27 | 0.17 |
|  | 2018 | Jo Y, 2018, INT J MOL SCI, V19, P0, DOI 10.3390/ijms19113684 | 27 | 0.09 |
|  | 2017 | Weller M, 2017, LANCET ONCOL, V18, P1373, DOI 10.1016/S1470-2045(17)30517-X | 27 | 0.04 |
|  | 2019 | Fabian D, 2019, CANCERS, V11, P0, DOI 10.3390/cancers11020174 | 27 | 0.02 |
|  | 2014 | Mrugala MM, 2014, SEMIN ONCOL, V41, PS4, DOI 10.1053/j.seminoncol.2014.09.010 | 27 | 0.01 |
|  | 2016 | Kim EH, 2016, ONCOTARGET, V7, P65125, DOI 10.18632/oncotarget.11372 | 27 | 0.01 |
|  | 2019 | Herrlinger U, 2019, LANCET, V393, P678, DOI 10.1016/S0140-6736(18)31791-4 | 25 | 0.05 |
|  | 2017 | Porat Y, 2017, JOVE-J VIS EXP, V0, P0, DOI 10.3791/55820 | 25 | 0.02 |
|  | 2016 | Kim EH, 2016, ONCOTARGET, V7, P62267, DOI 10.18632/oncotarget.11407 | 25 | 0 |
|  | 2014 | Miranda PC, 2014, PHYS MED BIOL, V59, P4137, DOI 10.1088/0031-9155/59/15/4137 | 24 | 0.02 |
|  | 2020 | Shi WY, 2020, J NEURO-ONCOL, V148, P489, DOI 10.1007/s11060-020-03540-6 | 24 | 0 |
|  | 2019 | Berkelmann L, 2019, SCI REP-UK, V9, P0, DOI 10.1038/s41598-019-43621-9 | 24 | 0 |
|  | 2016 | Voloshin T, 2016, INT J CANCER, V139, P2850, DOI 10.1002/ijc.30406 | 23 | 0.03 |
|  | 2014 | Giladi M, 2014, SEMIN ONCOL, V41, PS35, DOI 10.1053/j.seminoncol.2014.09.006 | 23 | 0.03 |
|  | 2013 | Davies AM, 2013, ANN NY ACAD SCI, V1291, P86, DOI 10.1111/nyas.12112 | 23 | 0.01 |
|  | 2015 | Wenger C, 2015, PHYS MED BIOL, V60, P7339, DOI 10.1088/0031-9155/60/18/7339 | 23 | 0.01 |
|  | 2019 | Neuhaus E, 2019, CANCERS, V11, P0, DOI 10.3390/cancers11010110 | 22 | 0 |
|  | 2014 | Giladi M, 2014, PANCREATOLOGY, V14, P54, DOI 10.1016/j.pan.2013.11.009 | 21 | 0.19 |
|  | 2020 | Lacouture ME, 2020, FRONT ONCOL, V10, P0, DOI 10.3389/fonc.2020.01045 | 21 | 0.02 |
|  | 2021 | Louis DN, 2021, NEURO-ONCOLOGY, V23, P1231, DOI 10.1093/neuonc/noab106 | 20 | 0 |
|  | 2019 | Kim EH, 2019, ONCOGENE, V38, P6630, DOI 10.1038/s41388-019-0882-7 | 20 | 0 |
|  | 2017 | Mehta M, 2017, CRIT REV ONCOL HEMAT, V111, P60, DOI 10.1016/j.critrevonc.2017.01.005 | 19 | 0.11 |
|  | 2017 | Wick W, 2017, NEW ENGL J MED, V377, P1954, DOI 10.1056/NEJMoa1707358 | 19 | 0.03 |
|  | 2020 | Voloshin T, 2020, CANCERS, V12, P0, DOI 10.3390/cancers12103016 | 19 | 0.01 |
|  | 2014 | Kanner AA, 2014, SEMIN ONCOL, V41, PS25, DOI 10.1053/j.seminoncol.2014.09.008 | 18 | 0.06 |
|  | 2015 | Chaudhry A, 2015, WORLD J SURG ONCOL, V13, P0, DOI 10.1186/s12957-015-0722-3 | 18 | 0.02 |
|  | 2020 | Bokstein F, 2020, FRONT ONCOL, V10, P0, DOI 10.3389/fonc.2020.00411 | 18 | 0.01 |
|  | 2016 | Bernard-Arnoux F, 2016, NEURO-ONCOLOGY, V18, P1129, DOI 10.1093/neuonc/now102 | 17 | 0.04 |
|  | 2018 | Lim M, 2018, NAT REV CLIN ONCOL, V15, P422, DOI 10.1038/s41571-018-0003-5 | 17 | 0.01 |
|  | 2020 | Reardon DA, 2020, JAMA ONCOL, V6, P1003, DOI 10.1001/jamaoncol.2020.1024 | 17 | 0.01 |
|  | 2017 | Trusheim John, 2017, CNS ONCOL, V6, P29, DOI 10.2217/cns-2016-0032 | 17 | 0 |
|  | 2017 | Zhu JJ, 2017, J NEURO-ONCOL, V135, P545, DOI 10.1007/s11060-017-2601-y | 16 | 0.01 |
|  | 2018 | Jo Y, 2018, CELL DEATH DISCOV, V4, P0, DOI 10.1038/s41420-018-0106-x | 16 | 0 |
|  | 2017 | Kesari Santosh, 2017, CNS ONCOL, V6, P185, DOI 10.2217/cns-2016-0049 | 16 | 0 |
|  | 2013 | Pless M, 2013, LUNG CANCER, V81, P445, DOI 10.1016/j.lungcan.2013.06.025 | 15 | 0.16 |
|  | 2015 | Ostrom QT, 2015, NEURO-ONCOLOGY, V16, P1, DOI 10.1093/neuonc/nou327 | 15 | 0.08 |
|  | 2009 | Kirson Eilon D, 2009, BMC MED PHYS, V9, P1, DOI 10.1186/1756-6649-9-1 | 15 | 0.05 |
|  | 2013 | Gilbert MR, 2013, J CLIN ONCOL, V31, P4085, DOI 10.1200/JCO.2013.49.6968 | 14 | 0.04 |
|  | 2021 | Mumblat H, 2021, LUNG CANCER, V160, P99, DOI 10.1016/j.lungcan.2021.08.011 | 14 | 0 |
|  | 2020 | Li X, 2020, IEEE T BIO-MED ENG, V67, P2594, DOI 10.1109/TBME.2020.2965883 | 14 | 0 |
|  | 2019 | Cloughesy TF, 2019, NAT MED, V25, P477, DOI 10.1038/s41591-018-0337-7 | 13 | 0.03 |
|  | 2019 | Lu GR, 2019, FRONT NEUROL, V10, P0, DOI 10.3389/fneur.2019.00042 | 13 | 0.01 |
|  | 2020 | Wen PY, 2020, NEURO-ONCOLOGY, V22, P1073, DOI 10.1093/neuonc/noaa106 | 13 | 0.01 |
|  | 2018 | Korshoej AR, 2018, PLOS ONE, V13, P0, DOI 10.1371/journal.pone.0201957 | 13 | 0 |
|  | 2020 | Tan AC, 2020, CA-CANCER J CLIN, V70, P299, DOI 10.3322/caac.21613 | 13 | 0 |
|  | 2014 | Stupp R, 2014, LANCET ONCOL, V15, P1100, DOI 10.1016/S1470-2045(14)70379-1 | 12 | 0.06 |
|  | 2018 | Liau LM, 2018, J TRANSL MED, V16, P0, DOI 10.1186/s12967-018-1507-6 | 12 | 0.01 |
|  | 2021 | Karanam NK, 2021, INT J RADIAT BIOL, V97, P1044, DOI 10.1080/09553002.2020.1837984 | 12 | 0 |
|  | 2014 | Wong ET, 2014, CANCER MED-US, V3, P592, DOI 10.1002/cam4.210 | 11 | 0.04 |
|  | 2016 | Wick W, 2016, NEURO-ONCOLOGY, V18, P303, DOI 10.1093/neuonc/now012 | 11 | 0.03 |
|  | 2020 | Ghiaseddin AP, 2020, CURR TREAT OPTION ON, V21, P0, DOI 10.1007/s11864-020-00773-5 | 11 | 0 |
|  | 2016 | Wenger C, 2016, INT J RADIAT ONCOL, V94, P1137, DOI 10.1016/j.ijrobp.2015.11.042 | 11 | 0 |
|  | 2012 | Fonkem E, 2012, EXPERT REV NEUROTHER, V12, P895, DOI 10.1586/ERN.12.80 | 11 | 0 |
|  | 2022 | Chen DJ, 2022, J CLIN INVEST, V132, P0, DOI 10.1172/JCI149258 | 11 | 0 |
|  | 2018 | Desjardins A, 2018, NEW ENGL J MED, V379, P150, DOI 10.1056/NEJMoa1716435 | 10 | 0.05 |
|  | 2011 | Pless M, 2011, EXPERT OPIN INV DRUG, V20, P1099, DOI 10.1517/13543784.2011.583236 | 10 | 0.01 |
|  | 2014 | Turner SG, 2014, WORLD J SURG ONCOL, V12, P0, DOI 10.1186/1477-7819-12-162 | 10 | 0 |
|  | 2017 | ORourke DM, 2017, SCI TRANSL MED, V9, P0, DOI 10.1126/scitranslmed.aaa0984 | 10 | 0 |
|  | 2021 | Blatt R, 2021, FRONT ONCOL, V11, P0, DOI 10.3389/fonc.2021.670809 | 10 | 0 |

**Table S3** The active or recruiting clinical trials of TTfields from www.clinicaltrials.gov.

| NCT Number | Title | Phases | Study Status | Conditions | Interventions |
| --- | --- | --- | --- | --- | --- |
| NCT03128047 | HUMC 1612: Optune NovoTTF-200A System | PHASE1 | Active, not yet recruiting | High Grade Glioma, Ependymoma | TTfields |
| NCT04221503 | Niraparib/TTFields in GBM | PHASE2 | Active, not yet recruiting | Recurrent Glioblastoma | Surgery, TTfields, and Niraparib |
| NCT03223103 | Safety and Immunogenicity of Personalized Genomic Vaccine and Tumor Treating Fields (TTFields) to Treat Glioblastoma | PHASE1 | Active, not yet recruiting | Newly Diagnosed Glioblastoma | TMZ, TTfields, and Tumor Antigen Vaccine |
| NCT05973903 | Lenvatinib, Pembrolizumab, and Tumor Treating Fields (TTFields) for Second-line Treatment of Glioblastoma | PHASE1\|PHASE2 | Not yet recruiting | Recurrent Glioblastoma | TTfields, Lenvatinib, and Pembrolizumab |
| NCT05004025 | Single Arm Trial of Tumor-Treating Fields in Combination With Nivolumab and Ipilimumab in Metastatic Uveal Melanoma | PHASE1 | Recruiting | Melanoma Uveal Metastasis | TTfields, Nivolumab, and Ipilimumab |
| NCT03203525 | Combination Chemotherapy and Bevacizumab With the NovoTTF-100L(P) System in Treating Participants With Advanced, Recurrent, or Refractory Hepatic Metastatic Cancer | PHASE1 | Recruiting | Advanced, Recurrent, or Refractory Hepatic Metastatic Cancer | TTfields, Bevacizumab, and Combined Chemotherapy |
| NCT03705351 | Tumor Treating Fields With Chemoradiation in Newly Diagnosed GBM | PHASE1 | Active, not yet recruiting | Newly Diagnosed Glioblastoma | TTfields, Temozolomide, and Radiotherapy |
| NCT03405792 | Study Testing The Safety and Efficacy of Adjuvant Temozolomide Plus TTFields (Optune®) Plus Pembrolizumab in Patients With Newly Diagnosed Glioblastoma (2-THE-TOP) (2-THE-TOP) | PHASE2 | Active, not yet recruiting | Newly Diagnosed Glioblastoma | TTfields, Temozolomide, and Pembrolizumab |
| NCT03033992 | Optune for Children With High-Grade Glioma or Ependymoma, and Optune With Radiation Therapy for Children With DIPG | Not Applicable | Active, not yet recruiting | Malignant Glioma, Ependymoma (Children) | TTfields, and Radiotherapy |
| NCT05746325 | Tumor Treating Fields for the Treatment of Leptomeningeal Metastases of the Spine in Patients With Breast Cancer | Not Applicable | Recruiting | Breast Cancer with Spine Leptomeningeal Metastases | TTfields |
| NCT03297125 | Assessment of Optune Therapy for Patients With Newly Diagnosed Glioblastoma Using Advanced MRI | OBSERVATIONAL | Recruiting | Newly Diagnosed Glioblastoma | TTfields |
| NCT05310448 | Tumor Treating Fields for the Treatment of Brainstem Gliomas | Not Applicable | Recruiting | Brainstem Gliomas | TTfields |
| NCT02441322 | Evaluating Therapeutic Response to Novo-TTF | Not Applicable | Active, not yet recruiting | Glioblastoma | TTfields |
| NCT05555342 | A Novel Method for Treating Lung Met w/Combo of Electric Fields & Rad Therapy: A Single-Arm | EARLY PHASE1 | Recruiting | Metastatic Lung Cancer | TTfields, and Radiotherapy |
| NCT02973789 | Effect of Tumor Treating Fields (TTFields) (150 kHz) Concurrent With Standard of Care Therapies for Treatment of Stage 4 Non-small Cell Lung Cancer (NSCLC) Following Platinum Failure (LUNAR) | PHASE3 | Active, not yet recruiting | Recurrent NSCLC | TTfields, and Immune checkpoint inhibitors or Docetaxel |
| NCT05583188 | Neoadjuvant Aliya™ PEF Soft Tissue Ablation With Systemic Therapy in Early-Stage Resectable NSCLC (VIGOR) | PHASE4 | Recruiting | NSCLC | Surgery, TTfields, Nivolumab, and Platinum |
| NCT04892472 | EF-36/Keynote B36: A Pilot, Randomized, Open-label Study of Tumor Treating Fields (TTFields, 150 kHz) Concomitant With Pembrolizumab for First Line Treatment of Advanced or Metastatic Non-small Cell Lung Cancer | PHASE2 | Recruiting | NSCLC | TTfields, Pembrolizumab |
| NCT05890872 | Aliya™ Pulsed Electric Fields (PEF) for Advanced Cancer (AFFINITY) | Not Applicable | Recruiting | Metastatic Lung Cancer or stage IV NSCLC | TTfields |
| NCT04474353 | Study of Tumor Treating Fields With Hypofractionated Chemoradiotherapy in Newly Diagnosed Glioblastoma | PHASE1 | Recruiting | Newly Diagnosed Glioblastoma | TTfields, Gadolinium, Temozolomide, Radiotherapy |
| NCT05653453 | Clinical Study of Tumor Treating Fields Combined With Gemcitabine and Albumin-bound Paclitaxel in the First-line Treatment of Locally Advanced Pancreatic Cancer | PHASE3 | Not yet recruiting | Locally Advanced Pancreatic Cancer | TTfields, Gemcitabine hydrochloride, Paclitaxel |
| NCT05341349 | Stereotactic Radiosurgery and Immune Checkpoint Inhibitors With NovoTTF-100M for the Treatment of Melanoma Brain Metastases | PHASE1 | Recruiting | Melanoma Brain Metastases | TTfields, Immune checkpoint inhibitors, and Radiotherapy |
| NCT03377491 | Effect of Tumor Treating Fields (TTFields, 150 kHz) as Front-Line Treatment of Locally-advanced Pancreatic Adenocarcinoma Concomitant With Gemcitabine and Nab-paclitaxel (PANOVA-3) | PHASE3 | Active, not yet recruiting | Locally Advanced Pancreatic Cancer | TTfields, Gemcitabine, and paclitaxel |
| NCT03194971 | NovoTTF Treatment Signatures in Glioblastoma Patients at Autopsy | OBSERVATIONAL | Recruiting | Glioblastoma | TTfields |
| NCT04717739 | TIGER PRO-Active - Daily Activity, Sleep and Neurocognitive Functioning Study | OBSERVATIONAL | Recruiting | Glioblastoma | TTFields |
| NCT04397679 | Partial Brain RT, Temozolomide, Chloroquine, and TTF Therapy for the Treatment of Newly Diagnosed Glioblastoma | PHASE1 | Recruiting | Newly Diagnosed Glioblastoma | TTFields, Radiotherapy, Temozolomide, and Chloroquine |
| NCT05086497 | WBSI Guided Personalized Delivery of TTFields | Not Applicable | Recruiting | Glioblastoma | TTFields under Whole Brain Spectroscopy Imaging Array Mapping Layout |
| NCT01892397 | Pilot Study of Optune (NovoTTF-100A) for Recurrent Atypical and Anaplastic Meningioma | Not Applicable | Active, not yet recruiting | Recurrent Atypical and Anaplastic Meningioma | TTFields |
| NCT02847559 | Optune Delivered Electric Field Therapy and Bevacizumab in Treating Patients With Recurrent or Progressive Grade 2 or 3 Meningioma | PHASE2 | Recruiting | Recurrent or Progressive Grade 2 or 3 Meningioma | TTFields, Bevacizumab |
| NCT04671459 | TTFields and Radiosurgery of Recurrent Glioblastoma +/- 18F-Fluoro-Ethyl-Thyrosine | PHASE2 | Active, not yet recruiting | Recurrent Glioblastoma | TTFields, Radiotherapy |
| NCT05764954 | A Study of Tumor-Treating Fields (TTFields) in People With Lung Adenocarcinoma | PHASE1 | Recruiting | Lung Adenocarcinoma | TTFields |
| NCT02831959 | Pivotal, Open-label, Randomized Study of Radiosurgery With or Without Tumor Treating Fields (TTFields) for 1-10 Brain Metastases From Non-small Cell Lung Cancer (NSCLC). | PHASE3 | Active, not yet recruiting | NSCLC Brain Metastases | TTFields, Radiotherapy |
| NCT05092373 | Tumor Treating Fields Therapy in Combination With Chemotherapy for the Treatment of Advanced Solid Tumors Involving the Abdomen or Thorax | PHASE1 | Recruiting | Advanced Solid Tumors Involving the Abdomen or Thorax | TTFields, Atezolizumab, Cabozantinib, and Paclitaxel |
| NCT05698264 | A Pilot Study to Understand the Impact of Therapy With Tumour Treating Fields (TTFields) in NSCLC | Not Applicable | Not yet recruiting | NSCLC | TTfields, and Immune checkpoint inhibitors |
| NCT03940196 | Effect of Tumor Treating Fields (TTFields, 200 kHz) Concomitant With Weekly Paclitaxel for the Treatment of Recurrent Ovarian Cancer (ENGOT-ov50 / GOG-3029 / INNOVATE-3) | PHASE3 | Active, not yet recruiting | Recurrent Ovarian Cancer | TTFields, Paclitaxel |
| NCT03642080 | MRI Predictors of Response to Tumor Treating Fields | OBSERVATIONAL | Recruiting | Glioblastoma | TTFields |
| NCT04471844 | Pivotal, Randomized, Open-label Study of Optune® (Tumor Treating Fields) Concomitant With RT & TMZ for the Treatment of Newly Diagnosed GBM (EF-32) | Not Applicable | Recruiting | Newly Diagnosed Glioblastoma | TTFields, Radiotherapy, and Temozolomide |
| NCT04605913 | Nab-Paclitaxel + Cisplatin + Gemcitabine + TTF in pt. w/ Metastatic PAC | PHASE1\|PHASE2 | Recruiting | Metastatic Pancreatic Adenocarcinoma | TTFields, Paclitaxel, Cisplatin, and Gemcitabine |
| NCT04223999 | Improving Tumor Treating Fields Treatment for Brain Cancer Patients With Skullremodeling Surgery (Neurosurgery) | PHASE2 | Recruiting | Recurrent Glioblastoma | TTFields plus Skullremodeling Surgery |
| NCT05679674 | Stereotactic Body Radiation and Tumor Treating Fields for Locally Advanced Pancreas Cancer | Not Applicable | Recruiting | Locally Advanced Pancreatic Cancer | TTFields, Radiotherapy |
| NCT05776524 | Study of Amplitude-Modulated Radiofrequency Electromagnetic Fields in Metastatic Pancreatic Cancer | PHASE2 | Recruiting | Metastatic Pancreatic Cancer | TTFields, Paclitaxel, Gemcitabine |
| NCT05661240 | Phase Ⅱ/​Ⅲ Clinical Study of Tumor Treating Fields （EFE-P100）Combined With Docetaxel in the Treatment of Stage IV Non-small Cell Lung Cancer Patients With Disease Progression After Platinum-based Chemotherapy and Anti-programmed Death 1（PD-1)/​Programmed Cell Death-Ligand 1(PD-L1) Antibody Treatment | PHASE2\|PHASE3 | Not yet recruiting | NSCLC | TTFields, Docetaxel |
